# Extracellularly secreted APE1/Ref-1 triggers apoptosis in triple-negative breast cancer cells via RAGE binding, which is mediated through acetylation

**DOI:** 10.18632/oncotarget.4345

**Published:** 2015-06-23

**Authors:** Yu Ran Lee, Ki Mo Kim, Byeong Hwa Jeon, Sunga Choi

**Affiliations:** ^1^ Research Institute of Medical Sciences, Department of Physiology, School of Medicine, Chungnam National University, Daejeon, 301747, Korea; ^2^ Cancer Research Team, Korean Medicine Based Herbal Drug Research Group, Herbal Medicine Research Division, Korea Institute of Oriental Medicine, (KIOM), Daejeon, 305811, Korea

**Keywords:** triple-negative breast cancer, acetylation, apoptosis, RAGE, APE1/Ref-1

## Abstract

The present study evaluated the mechanism of apoptosis caused by post-translational modification, hyperacetylation in triple-negative breast cancer (TNBC) cells. We previously showed that trichostatin A (TSA) induced secretion of acetylated apurinic apyrimidinic endonuclease 1/redox factor-1 (Ac-APE1/Ref-1). This is the first report showing that Ac-APE1/Ref-1 initiates apoptosis in TNBC cells by binding to the receptor for advanced glycation end products (RAGE). The functional significance of secreted Ac-APE1/Ref-1 was studied by induction of intracellular hyperacetylation through co-treatment with acetylsalicylic acid and TSA in MDA-MB-231 cells. In response to hyperacetylation, secretion of Ac-APE1/Ref-1 in vesicles was observed, resulting in significantly decreased cell viability and induction of apoptosis with increased expression of RAGE. The hyperacetylation-induced apoptosis was similar in two other TNBC cell lines: BT-459 and MDA-MB-468. Therefore, hyperacetylation may be a therapeutic target for treatment of TNBCs. This study introduces a novel paradigm whereby post-translational modification induces apoptotic cell death in breast cancer cells resistant to standard chemotherapeutic agents through secretion of auto- or paracrine molecules such as Ac-APE1/Ref-1.

## INTRODUCTION

Breast cancer is the most prevalent cancer in women worldwide. The incidence of breast cancer is increasing and 1.7 million new cases were diagnosed in 2012 [1]. Estrogen and progesterone are important in breast cancers expressing the estrogen receptor (ER) and progesterone receptor (PR) [2, 3]. Hormone-related factors that reflect exposure to estrogen and progesterone can be derived from family history, atypical hyperplasia of the breast, early age at menarche, nulliparity, delayed childbearing, and late menopause [4, 5]. These factors are associated with an increased incidence of breast cancers that express ER and PR, but not with breast cancers lacking expression of these receptors [6, 7]. Several clinical studies have reported that ER/PR-positive breast cancers respond effectively to endocrine therapies [8–10]. Indeed, in clinical trials, agents that block the ER, such as tamoxifen and aromatase inhibitors, increased patient survival [11]. Cells in 20% to 30% of breast cancers express the human epidermal growth factor receptor (HER2/neu). Breast cancers with HER2/neu are much more aggressive and fast-growing and can be treated with Herceptin or Tykerb, which inhibit HER2/neu-mediated signaling [12, 13].

Triple-negative (ER, PR and HER2/neu) breast cancers (TNBCs) are a heterogeneous basal-like subset defined by gene expression profiling that demonstrates the absence of therapeutic target receptors [14]. This subtype has a higher histological grade, appears in a disproportionately high number of metastatic cases, and exhibits earlier recurrence at distant sites, resulting in poor prognoses [15–17]. TNBC patients do not benefit from current receptor-targeted treatments. Therefore, the patients must rely on limited therapeutic options, primarily chemotherapy, even though the cancer cells have become resistant to the treatment [18]. New targeted therapies are an urgent unmet medical need for this patient population.

Post-translational modifications (PTMs) including acetylation, phosphorylation, and methylation are primarily responsible for the regulation of gene expression. PTMs can induce a dynamic interplay between survival pathways and apoptosis by sensitizing cancer cells to therapeutic agents. Thus, agents causing PTMs can be used to primarily treat some chemotherapy-resistant cancers [19–22]. Acetylation is a major PTM of eukaryotic cellular proteins and emerges as a critical factor in regulating cell cycle arrest and apoptosis [23]. Several agents affecting acetylation, particularly histone deacetylase inhibitors (HDACi), have potent anti-tumor activity *in vivo* suggesting their usefulness as cancer therapeutic agents [24]. PTMs at specific residues are involved in the sequestration of proteins to cellular organelles. The nuclear-cytoplasmic shuttling of proteins has direct implications for dynamic changes in the role of the translocated protein.

Bonaldi and co-workers demonstrated that secretion of high mobility group box 1 (HMGB1) (a potent cytokine that triggers inflammatory mediators) by macrophages is mediated through acetylation that prevents nuclear reentry and allows packaging into secretory vesicles [25]. Extracellularly, HMGB1 shows anti-tumor activity as a chemoattractant, activating the innate immune system [26]. Recently, we reported that nuclear apurinic apyrimidinic endonuclease 1/redox factor-1 (APE1/Ref-1) was acetylated at lysine residues K6 and K7, translocated into the cytoplasm in response to treatment with an HDACi (Trichostatin A [TSA]), and then secreted extracellularly. The amount of acetylated APE1/Ref-1 (Ac-APE1/Ref-1) was determined even though the role of secreted Ac-APE1/Ref-1 was unknown [27].

Our interest in the role of secreted Ac-APE1/Ref-1 stemmed from studies documenting the involvement of extracellular secretory proteins in signal transduction mediated by autocrine and/or paracrine mechanisms [28–30]. Signaling molecules that stimulate specific receptors can initiate a cascade of intracellular reactions leading to cellular responses. In the current study, we first propose that the receptor for advanced glycation end products (RAGE) is a target for extracellular Ac-APE1/Ref-1. RAGE is a transmembrane receptor in the immunoglobulin superfamily and is activated by binding multiple distinct ligands such as AGEs, amyloid β-peptide, HMGB-l, and S100/calgranulins [31]. This broad ligand repertoire results from the ability of RAGE to recognize tertiary structures rather than a unique primary structure within the ligand [31]. Moreover, activated RAGE is involved in the pathogenesis of several diseases including atherosclerosis, Alzheimer's disease, arthritis, and diabetes [31]. Involvement of RAGE in cancer cell proliferation, metastasis, and invasion has been reported, indicating that RAGE is a potential therapeutic target [32]. Some reports have shown different cellular responses through RAGE activation by different ligands, suggesting distinct intracellular roles. For example, RAGE activation induced cell death via p-38 MAPK/ERK signaling through binding with HMGB-1 in neuronal cells [33]. In addition, extra-nuclear translocation of acetylated HMGB-1 was accompanied by phosphorylation of p38 MAPK in genistein-treated cervical cancer HeLa cells [34].

The current study investigated the functional significance of secreted Ac-APE1/Ref-1 in hyperacetylated TNBC, MDA-MB-231 cells. We also used two other TNBC cell lines (MDA-MB-468 and BT-549), and RAGE-overexpressing or -knockdown MDA-MB-231 cells to evaluate the central role of RAGE in the transduction of apoptotic signals. The present study provides compelling experimental evidence to indicate that the stimulation of apoptosis by the binding of secreted Ac-APE1/Ref-1 with RAGE is essential for the death of hyperacetylated TNBC cells.

## RESULTS

### Hyperacetylation by treatment with acetylsalicylic acid (ASA) and TSA causes different types of cell death in the MCF-7 and MDA-MB-231 human breast cancer cell lines

**Figure 1:**
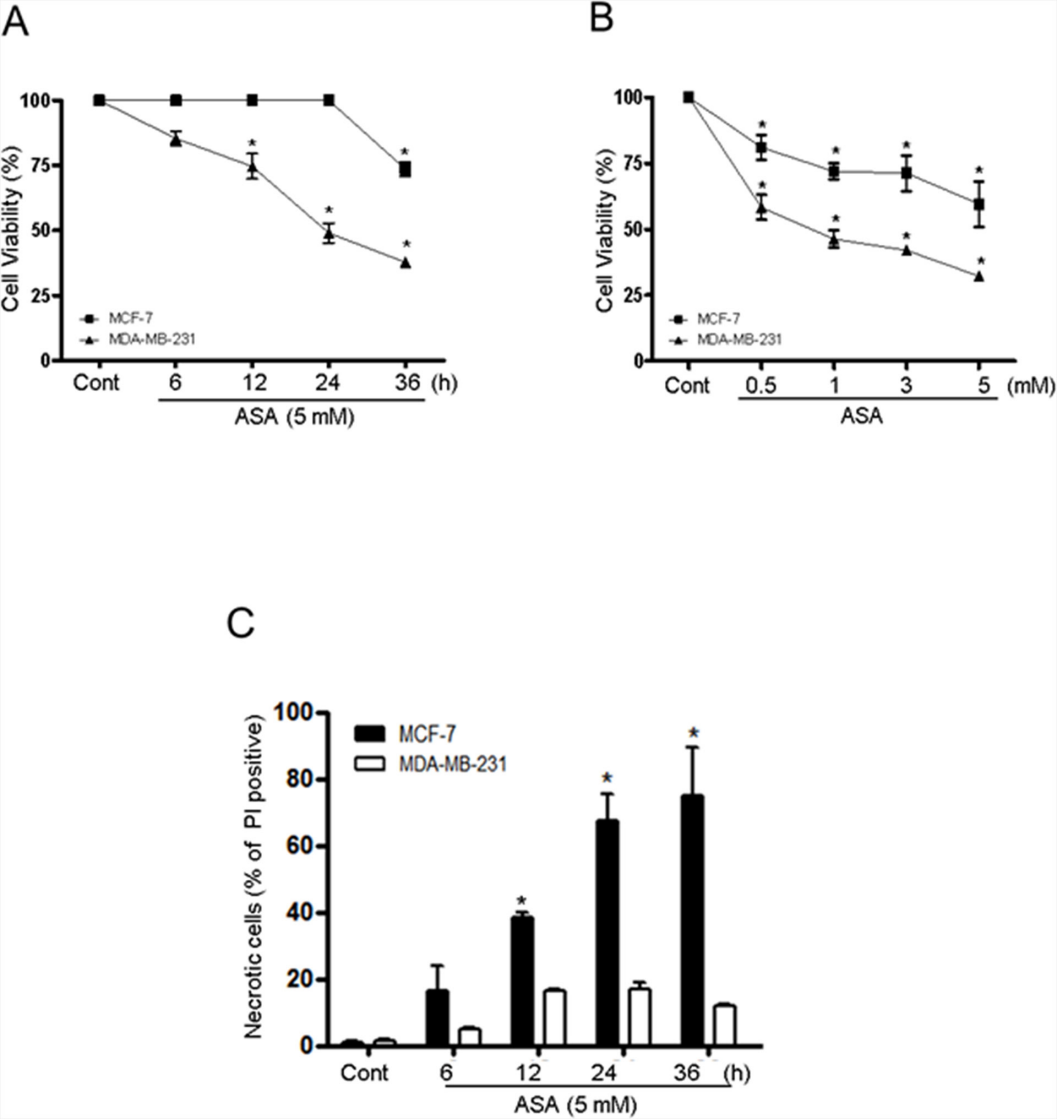
Hyperacetylation decreased the viability of triple-negative breast cancer MDA-MB-231 and ER/PR-positive breast cancer MCF-7 cells **A** and **B.** MDA-MB-231 and MCF-7 cells were pretreated with TSA (0.1 μM) for 1 h, and then with SA (control) or 5 mM ASA for the indicated times and concentrations. Cell viability was determined by an automatic cell counter (ADAM-MC) using an AccuChip4x kit. C. Cells were treated with SA (control) or 5 mM ASA for the indicated times and stained with PI without using a permeabilizing detergent. PI-positive cells were analyzed using an automatic cell counter. *Columns*, mean (*n* = 3); *bars*, SE. *, *P* < 0.05, significantly different from SA-treated control cells by one-way ANOVA followed by Dunnett's test. Similar results were observed in replicate experiments.

Previous studies showed that treatment of cancer cells with ASA or TSA caused cell death *in vivo* and *in vitro* [35–37] resulting from the acetylation and subsequent functional alteration of multiple cellular proteins associated with the cell cycle, proliferation, differentiation, and death [37, 38]. However, the mechanism leading to cell death in response to acetylation is poorly defined. To investigate the potential for cell death to be regulated by acetylation, we determined the effect of co-treatment with ASA and TSA on cell viability in MDA-MB-231 and MCF-7 human breast cancer cell lines. The viability of MDA-MB-231 and MCF-7 cells pretreated for 1 h with 0.1 μM TSA was significantly decreased after exposure to ASA (Fig. 1A). However, the two breast cancer cell lines showed different temporal patterns of cell death. The viability of MDA-MB-231 cells decreased gradually with time after exposure to 5 mM ASA, and viability was 68% lower than that of SA-treated control cells at 36 h (Fig. 1B). In contrast, MCF-7 cells remained unaffected until a sharp decline of 35.8% in viability occurred after 24 h. The viability of MCF-7 cells was slightly decreased as ASA concentrations increased compared with MDA-MB-231 cells (Fig. 1A and 1B). Neither SA nor TSA alone caused cell death (data not shown). PI staining without detergent permeabilization was used to determine whether the ASA-mediated decrease in the viability of MCF-7 cells was caused by non-apoptotic cell death [39]. ASA treatment increased the number of PI- stained MCF-7 cells compared to MDA-MB-231 cells, indicating plasma membrane rupture of MCF-7 cells (Fig. 1C).

**Figure 2:**
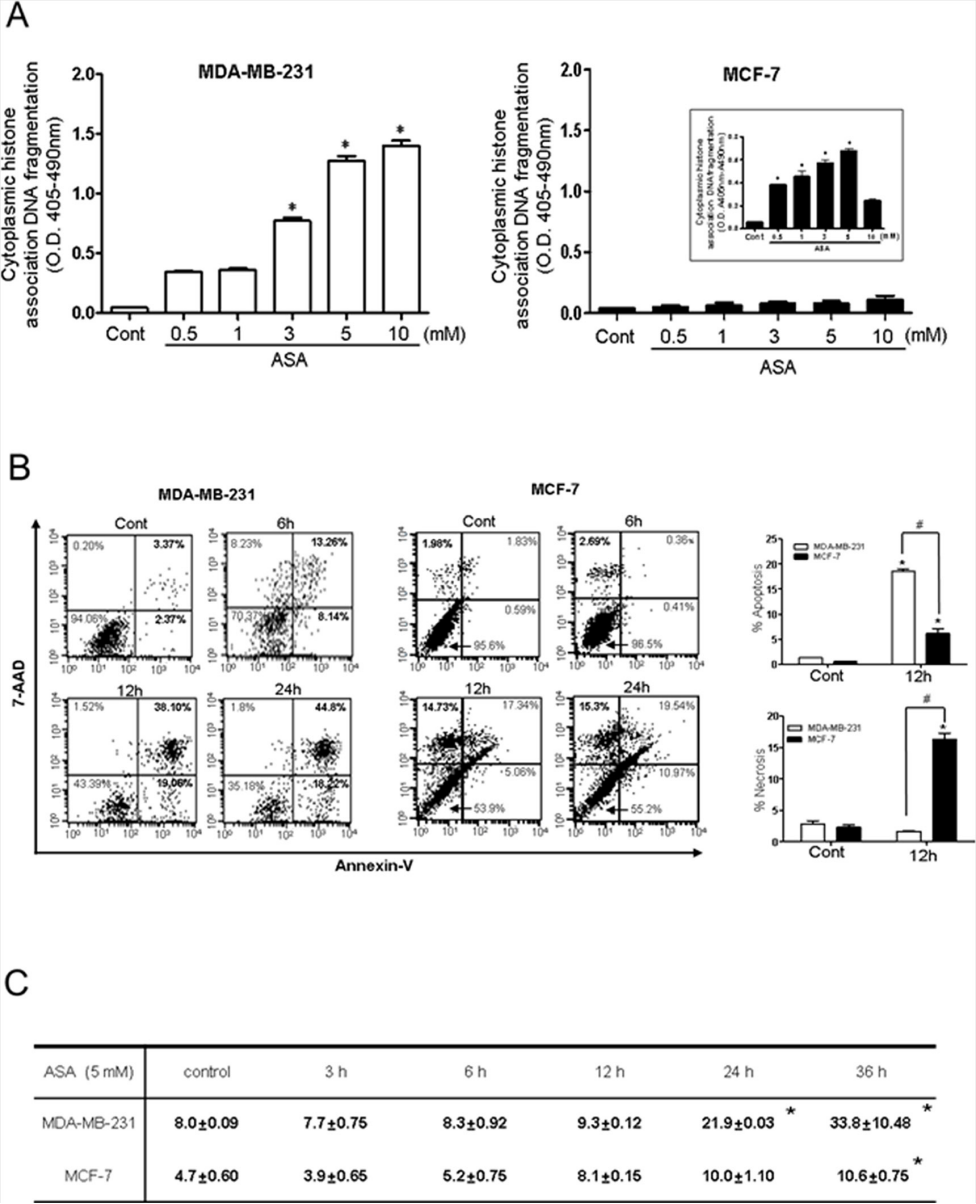
Hyperacetylation induced apoptosis in MDA-MB-231 cells but not in MCF-7 cells **A.** Apoptosis in MDA-MB-231 (left) and MCF-7 (right) cells was assessed by quantification of cytoplasmic histone-associated DNA fragmentation. Cells were exposed to the indicated concentrations of SA (control) or ASA for 36 h in the presence of TSA (0.1 μM). Both floating and adherent cells were separately collected and processed for the DNA fragmentation assay. The inset shows DNA fragmentation resulting from to non-apoptotic cell death (necrosis) in floating MCF-7 cells. *Columns*, mean (*n* = 3); *bars*, SE. *, *P* < 0.05, significantly different from SA-treated control cells by one-way ANOVA followed by Dunnett's test. **B.** A flow cytometry-based Annexin V/7-aminoactinomycin D (7-AAD) staining assay was used to determine apoptosis and necrosis. Apoptotic and necrotic cells were identified as Annexin V^+^/7-AAD^−^ (lower right quadrant) and Annexin V^−^/7-AAD^+^ (upper left quadrant), respectively. Quantification of the FACS results revealed that ASA treatment for 12 h significantly increased the Annexin V^+^/7-AAD^−^ population in MDA-MB-231 cells (left panel), wherease it increased the Annexin V^−^/7-AAD^+^ population (middle panel) in MCF-7 cells. *Column* mean (*n* = 3); *bars*, SE.*, ^#^, *P* < 0.05, significantly different compared with SA-treated control or between the indicated groups by one-way ANOVA followed by Bonferroni's multiple comparison test. **C.** Percentage of MDA-MB-231 and MCF-7 cells in the sub-G_0_ phase of the cell cycle following treatment with SA (control) or ASA (5 mM) for 36 h in the presence of TSA (0.1 μM). Similar results were observed in replicate experiments.

As shown in Fig. 2A, hyperacetylation-mediated apoptosis was examined by analysis of cytoplasmic histone-associated DNA fragmentation, a well-accepted technique for detection of apoptotic cell death in both MDA-MB-231 and MCF-7 cells. ASA treatment resulted in a concentration-dependent increase in the number of apoptotic cells in MDA-MB-231 cells, (Fig. 2A, left). DNA fragmentation in MDA-MB-231 cells treated with 5 mM ASA for 36 h was increased by approximately 13-fold compared with that observed in the SA-treated control cells. After treatment of MCF-7 cells with ASA, attached cells were carefully separated from the culture supernatant, which may contain DNA fragments as a result of traumatic cell death. Marginal DNA fragmentation was observed in the attached cells at higher concentrations of ASA (Fig. 2A right). The amount of nucleosomes released from the cells in the culture supernatant was increased by 7-fold in ASA-treated cells in a concentration-dependent manner, further indicating non-apoptotic cell death (Fig. 2A, right panel, inset). The different type of cell death at early stage was confirmed using Annexin V/7-AAD staining in FACS analysis. As shown in Fig. 2B, the Annexin V^+^/7-AAD^−^ cell population, indicating apoptotic cell death after ASA treatment was significantly increased in MDA-MB-231 cells. Consistent with the results of the DNA fragmentation assay, exposure of MCF-7 cells to ASA resulted in an increased Annexin V^−^/7-AAD^+^ population, indicating early necrotic cells.

In addition, exposure of MDA-MB-231 cells to 5 mM ASA enriched the sub G_0_/G_1_ fraction in a time-dependent manner while MCF-7 cells were resistant to ASA treatment until 24 h (Fig. 2C). At this time, a comparable number of PI-positive MCF-7 cells in the sub G_0_/G_1_ fraction could be detected by flow cytometry. Collectively, these results indicate that hyperacetylation mediated by ASA caused apoptotic cell death in MDA-MB-231 cells in a time- and concentration-dependent manner. Because ASA treated-MCF-7 cells showed necrotic cell death, we further explored the potential molecular mechanisms involved in hyperacetylation using TNBC, MDA-MB-231 cells.

### Hyperacetylated MDA-MB-231 cells secrete Ac-APE1/Ref-1 through vesicle formation

**Figure 3:**
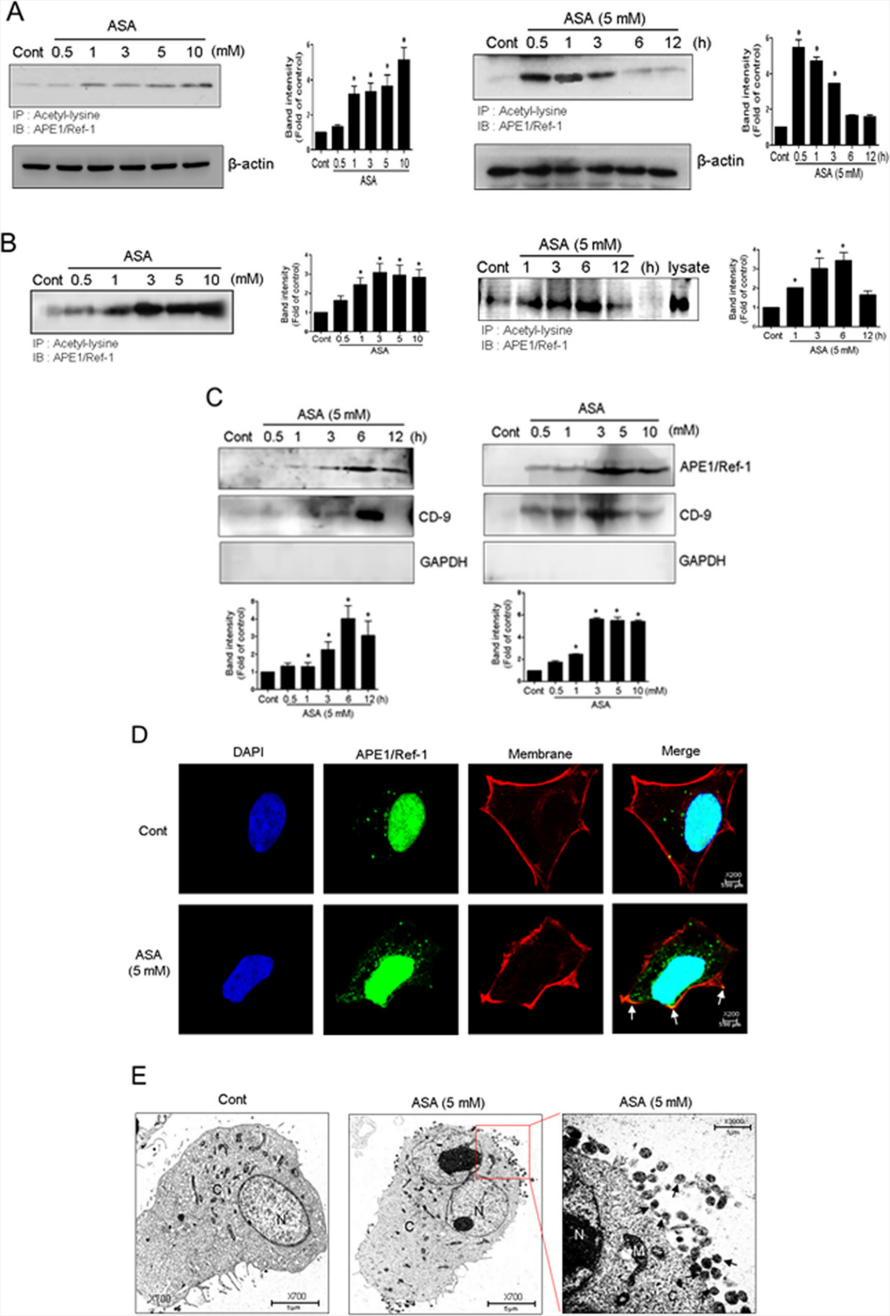
Secretion of Ac-APE1/Ref-1 by vesicle formation in response to hyperacetylation MDA-MB-231 cells were treated with SA (control) or ASA at the indicated concentrations and times in the presence of TSA (0.1 μM). The cells were harvested and conditioned medium was collected. Whole cell lysates **A.** and the conditioned media **B.** were immunoprecipitated with anti-acetyl-lysine followed by anti-APE1/Ref-1 antibodies. **C.** Vesicles in the conditioned medium were isolated by centrifugation at 100, 000 *g*. The resultant pellet was analyzed using anti-APE1/Ref-1, CD-9, or GAPDH antibodies. In A to C, *columns*, mean (*n* = 2–3); *bars*, SE. *, *P* < 0.05, significantly different from SA-treated control cells by one-way ANOVA followed by Dunnett's test. These experiments were performed with similar results. Representative blots are shown. **D.** Representative 3D confocal microscopic image of Ac-APE1/Ref-1. The plasma membrane was stained in red using CellMask deep red dye before cell fixation. Cells were immunostained with Alexa 488-conjugated anti-APE1/Ref-1 antibody. Green, red, and blue fluorescence indicates APE1/Ref-1, membrane, and nucleus, respectively. Exposure to ASA (5 mM) for 3 h caused a significant relocation of APE1/Ref-1 to the plasma membrane (yellow-orange, white arrowheads). **E.** Immunogold electron microscopic analysis of SA (control) or ASA-treated MDA-MB-231 cells in the presence of TSA. The low-magnification overview shows whole cells. Vesicles are present in the area around the nucleus near the plasma membrane in ASA-treated MDA-MB-231 cells compared with SA-treated control cells. Gold-labeled Ac-APE1/Ref-1 (black arrowheads) is seen in vesicles near the plasma membrane.

We previously showed that acetylation of cellular proteins by inhibiting HDAC induced the extracellular release of APE1/Ref-1 in human epithelial kidney 293T cells [27]. Because co-treatment with ASA and TSA also caused protein hyperacetylation and decreased the viability of MDA-MB-231 cells, we hypothesized that the apoptotic death of these cells may be associated with secreted Ac-APE1/Ref-1. Hyperacetylation of whole cellular protein was increased in a concentration- and time-dependent manner in response to ASA and TSA co-treatment (data not shown). The acetylation of APE1/Ref-1 was saturated within 1 h after ASA treatment and then gradually declined (Fig. 3A). However, the secretion of Ac-APE1/Ref-1 was concentration-dependent and increased up to 6 h after ASA treatment (Fig. 3B). The amount of secreted Ac-APE1/Ref-1 was related to the level of intracellular protein acetylation. These results indicated that the acetylated form of APE1/Ref-1 was sustained for 6 h under hyperacetylated conditions.

To address the possibility that vesicle formation was involved in the release of Ac-APE1/Ref-1 [40], vesicles were prepared from culture supernatants of ASA-treated MDA-MB-231 cells. Levels of Ac-APE1/Ref-1 were increased in the vesicle-enriched fraction in a concentration- and time-dependent manner (Fig. 3C, 3D). Compared with acetylation of intracellular proteins, the secretion of vesicles containing Ac-APE1/Ref-1 was relatively retarded by 6 h. Vesicle enrichment was confirmed using the anti-CD-9 antibody, which is a genearl molecular marker for exosomes (Fig. 3C). Formation of vesicles containing Ac-APE1/Ref-1 was confirmed by immunocytochemistry. Green fluorescent APE1/Ref-1 in the SA-treated control cell was distributed at the margin of the cytoplasmic membrane, whereas vesicles positive for Ac-APE1/Ref-1 were fused with the plasma membrane in ASA-treated cell as evidenced by yellow-orange staining (Fig. 3D, white arrow). As shown in Fig. 3E, hyperacetylated MDA-MB-231 cell released vesicles containing Ac-APE1/Ref-1 at the margin of the plasma membrane, in contrast to SA-treated control cell. Moreover, gold particle-labeled Ac-APE1/Ref-1 was clearly observed within the secreted vesicles (black arrow). Collectively, these results implicate secreted Ac-APE1/Ref-1 in apoptotic signaling occurring in response to hyperacetylation.

### Hyperacetylation upregulates RAGE in the plasma membrane

**Figure 4:**
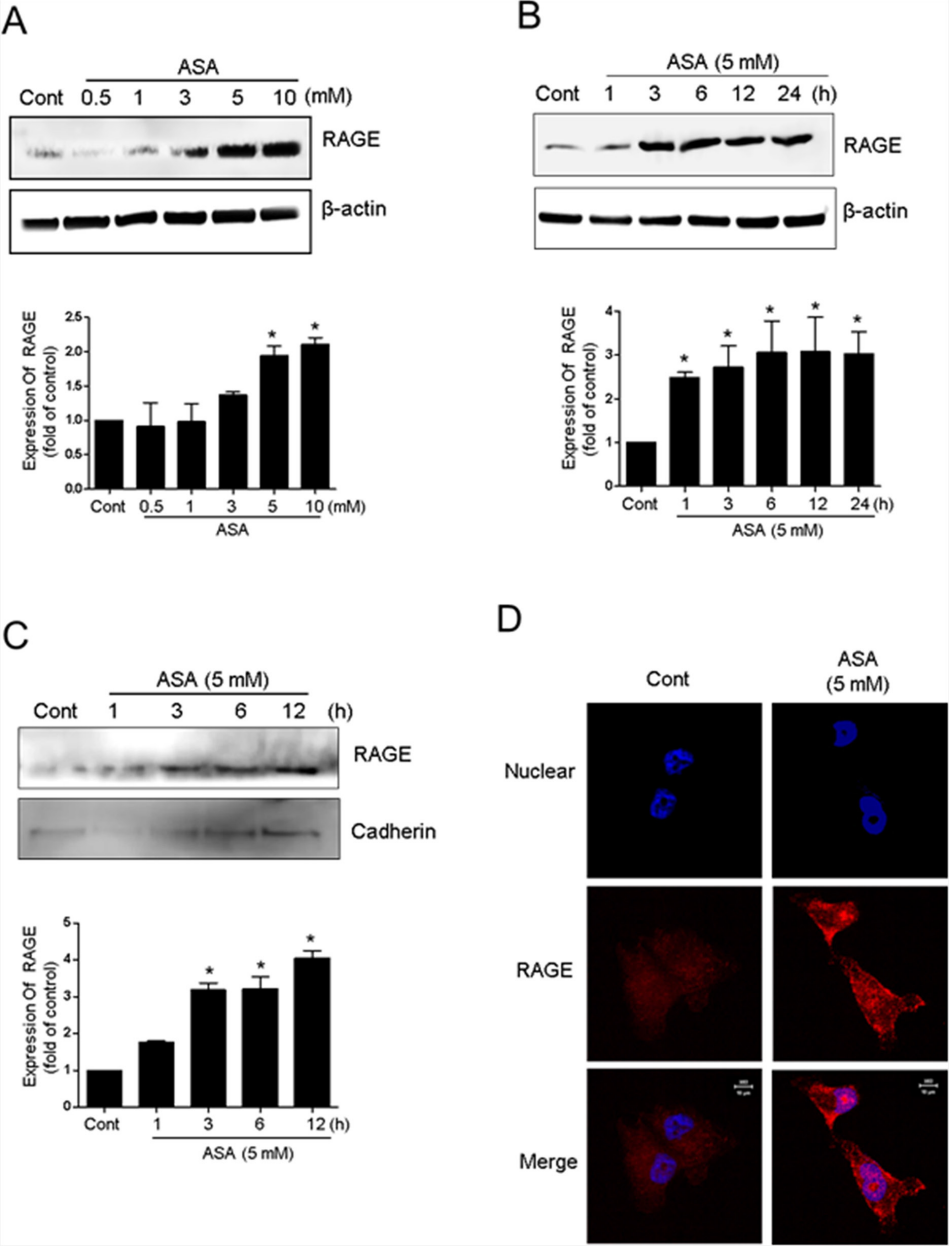
Upregulation of RAGE in response to hyperacetylation MDA-MB-231 cells were treated with SA (control) or ASA at the indicated concentrations and times in the presence of TSA (0.1 μM). Whole cell lysates **A** and **B.** or membrane fractions **C.** from the hyperacetylated cells were prepared. Immunoblotting for RAGE expression was performed using an anti-RAGE antibody. Blots were stripped and re-probed with anti-actin or cadherin antibodies to correct for differences in protein loading. In A to C, *columns*, mean (*n* = 2–3); *bars*, SE. *, *P* < 0.05, significantly different from SA-treated control cells by one-way ANOVA followed by Dunnett's test. The experiment was repeated with similar results. The results of representative blots are shown. **D.** 3D confocal immunofluorescence images of RAGE in MDA-MB-231 cells treated with SA (control) or ASA for 6 h. Cells were fixed without permeabilization and stained with DAPI and an anti-RAGE antibody. Optical slices (0.5 μm) were examined using a 63 × oil immersion objective.

Some stimulators of cell death are closely associated with membrane receptors that trigger and coordinate the intracellular signaling cascade. Because RAGE serves as a primary extracellular membrane receptor for multiple ligands that have a common tertiary structure, including alkylated secretory proteins [31], it is plausible that RAGE can also recognize the acetyl moiety on lysine residues of Ac-APE1/Ref-1. Exposure of MDA-MB-231 cells to ASA concentrations that decreased viability and increased apoptosis also increased RAGE expression in a concentration- and time-dependent manner (Fig. 4A, 4B). Hyperacetylation-mediated upregulation of RAGE was also evident in the cytoplasmic membrane fraction, as confirmed using an anti-cadherin antibody 3 h after ASA treatment (Fig. 4C). Using immunocytochemistry, RAGE was observed near the nucleus in SA-treated control cells. In contrast, RAGE in ASA-treated MDA-MB-231 cells was mostly found in the plasma membrane (Fig. 4D), which suggests that binding may occur between RAGE and Ac-APE1/Ref-1.

### Secreted Ac-APE/Ref-1 binds directly to RAGE

**Figure 5:**
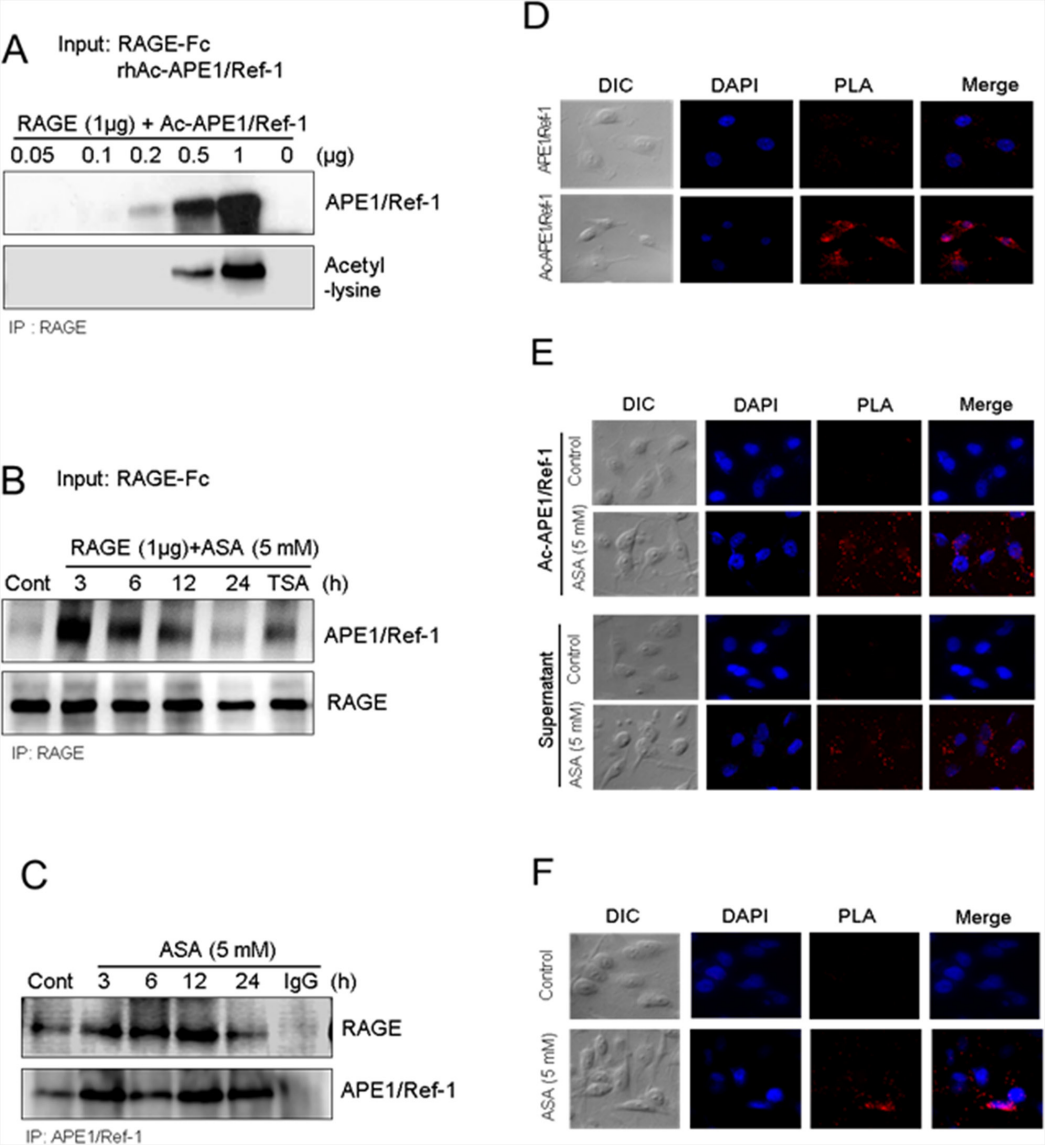
Secreted Ac-APE1/Ref-1 bound to RAGE in response to hyperacetylation **A.** Direct binding between RAGE-Fc and rh Ac-APE1/Ref-1, **B.** between RAGE-Fc and Ac-APE1/Ref-1 from conditioned medium, and **C.** between RAGE in the membrane fraction and secreted Ac-APE1/Ref-1. Binding was observed by co-immunoprecipitation with anti-APE1/Ref-1 or RAGE antibodies followed by anti-RAGE or APE1/Ref-1 antibodies, respectively. The binding interaction between Ac-APE1/Ref-1 and RAGE in the plasma membrane was visualized using with a Duolink II PLA system with primary anti-APE1/Ref-1 and anti-RAGE antibodies. **D.** RAGE^OV^ MDA-MB-231 cells were incubated with rh APE1/Ref-1 (upper panels) or rh Ac-APE1/Ref-1 (lower panels). **E.** MDA-MB-231 cells were treated with ASA and then further incubated with rh Ac-APE1/Ref-1 (upper panels) or the medium containing secreted Ac-APE1/Ref-1 (lower panels) rather than the normal culture supernatant. **F.** MDA-MB-231 cells were treated with SA (upper panels) or ASA (lower panels). Cells were then subjected to Duolink II PLA. The PLA-specific fluorescence which represents the Ac-APE1/Ref-1-RAGE signals, and the DAPI nucleic staining were colored red and blue, respectively. The experiment was repeated multiple times with similar results; data shown are from a representative experiment.

Because secretion of Ac-APE1/Ref-1 and increased RAGE expression were observed in response to hyperacetylation, we investigated whether Ac-APE1/Ref-1 could bind to RAGE and induce cell death in MDA-MB-231 cells. We tested the binding ability by co-immunoprecipitation of RAGE and Ac-APE1/Ref-1; binding was assessed between the recombinant proteins, Ac-APE1/Ref-1 and RAGE-Fc, between secreted Ac-APE1/Ref-1 in the culture medium and RAGE-Fc, and between secreted Ac-APE1/Ref-1 in the culture medium and RAGE in the membrane fraction. Following preparation of rh Ac-APE1/Ref-1 by incubation with ASA solution, rh Ac-APE1/Ref-1 formed a tight immune complex with rh RAGE-Fc (Fig. 5A). Secreted Ac-APE1/Ref-1 in the culture supernatant of ASA-treated MDA-MB-231 cells bound to RAGE-Fc (Fig. 5B). Binding of secreted APE1/Ref-1 with RAGE in the plasma membrane fraction was confirmed (Fig. 5C), and the interaction was significantly increased following acetylation (Fig. 3B).

Duolink II cell-based fluorescence PLA was used to visualize the direct binding of Ac-APE1/Ref-1 and RAGE [41]. RAGE^OV^ or hyperacetylated MDA-MB-231 cells were incubated with rh APE1/Ref-1, rh Ac-APE1/Ref-1, or culture supernatant, and then with a mixture of anti-RAGE and anti-acetyl-lysine antibodies. The interaction signal was amplified by further incubation with respective minus or plus PLA fluorescent probes. The binding of rh Ac-APE1/Ref-1 in RAGE^OV^ cells was visible as numerous red spots (Fig. 5D). In contrast, treatment with rh APE1/Ref-1 showed only faint background signals. When PLA was performed for ASA-treated MDA-MB-231 cells, stronger signals for rh Ac-APE1/Ref-1 or culture supernatant were observed compared to SA-treated control cells (Fig. 5E). The plasma membrane of MDA-MB-231 cells treated with ASA exhibited numerous PLA spots, in contrast to SA-treated control cells (Fig. 5F), which indicated close proximity between RAGE and Ac-APE1/Ref-1 (< 40 nm). Collectively, these results demonstrated that RAGE and secreted Ac-APE1/Ref-1 are indirectly or directly interacting with each other in hyperacetylated cells, although a direct interaction is more likely given our other data.

### RAGE is involved in apoptosis signaling through Ac-APE1/Ref-1

**Figure 6:**
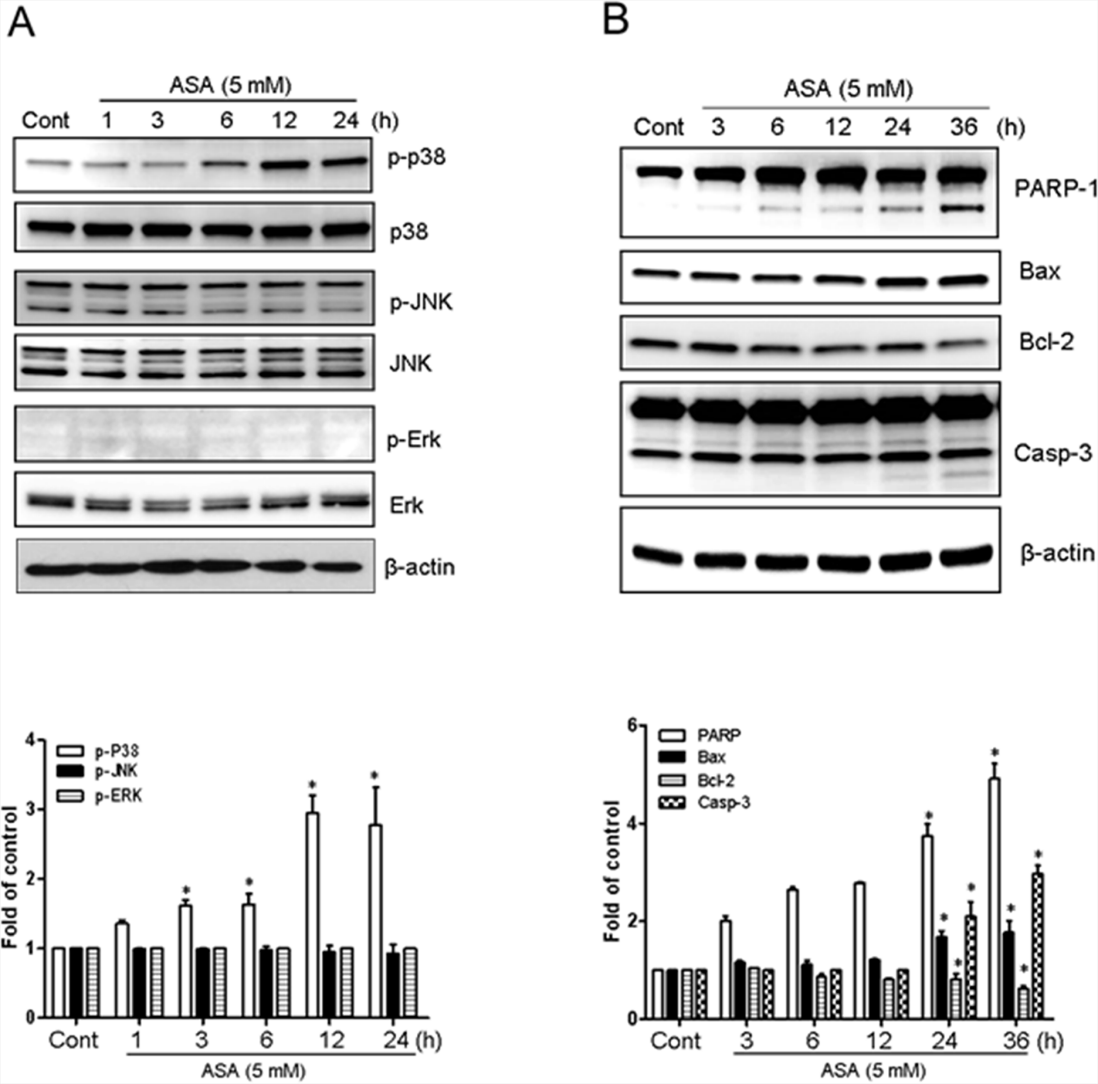
Hyperacetylation-activated apoptosis in MDA-MB-231 cells involved the phosphorylation of p38 MAPK Cell lysates were obtained from MDA-MB-231 cells treated with SA (control) or 5 mM ASA for the indicated times. **A.** Immunoblotting for phospho-JNK, phospho-Erk1/2, and phospho-p38 MAPK. **B.** Immunoblotting for PARP-1, Bax, Bcl-2, and caspase-3. The blots were stripped and reprobed with anti-JNK, -ERK, -p38, or -actin antibodies to ensure equal protein loading. Immunoblotting for each protein was performed two or more times by using independently prepared lysates with similar results. Fold changes in phosphorylated vs. total protein or the levels of apoptosis markers relative to control are shown for each time point. *Columns*, mean (*n* = 2–3); *bars*, SE. *, *P* < 0.05, significantly different from SA-treated control cells by one-way ANOVA followed by Dunnett's test. Data shown are representative of replicate experiments with similar results.

When stimulated by ligand binding, RAGE activates multiple signaling pathways, including MAPK [33, 42]. To elucidate the mechanism by which Ac-APE1/Ref-1 binding-mediated apoptosis stimulates RAGE, the effect on MAPK phosphorylation was determined. Treatment of MDA-MB-231 cells with 5 mM ASA resulted in no change in total p38 MAPK but increased its phosphorylation at Tyr^182^ by approximately 62% at 3 h, and this effect persisted to 24 h after treatment, compared with SA-treated controls (Fig. 6A). Phosphorylation of p38 MAPK occurred after secretion of Ac-APE1/Ref-1 into the medium (Fig. 3B) and the increase of RAGE in the plasma membrane (Fig. 4C). Exposure of MDA-MB-231 cells to ASA did not activate JNK1/2 or ERK.

Expression of the pro-apoptotic Bax protein was increased by 1.5 to 2 fold in ASA-treated MDA-MB-231 cells, whereas levels of the anti-apoptotic protein Bcl-2 were decreased (Fig. 6B). ASA treatment also caused time-dependent cleavage of PARP-1 by activation of caspase-3 (p17 cleaved form). Together, these results suggest that activation of p38 MAPK, and apoptosis signaling through the Bcl-2 family of proteins were mediated by stimulation of RAGE in response to binding with Ac-APE1/Ref-1.

### Increased RAGE expression induces apoptosis in Ac-APE1/Ref-1-stimulated TNBC cells

**Figure 7:**
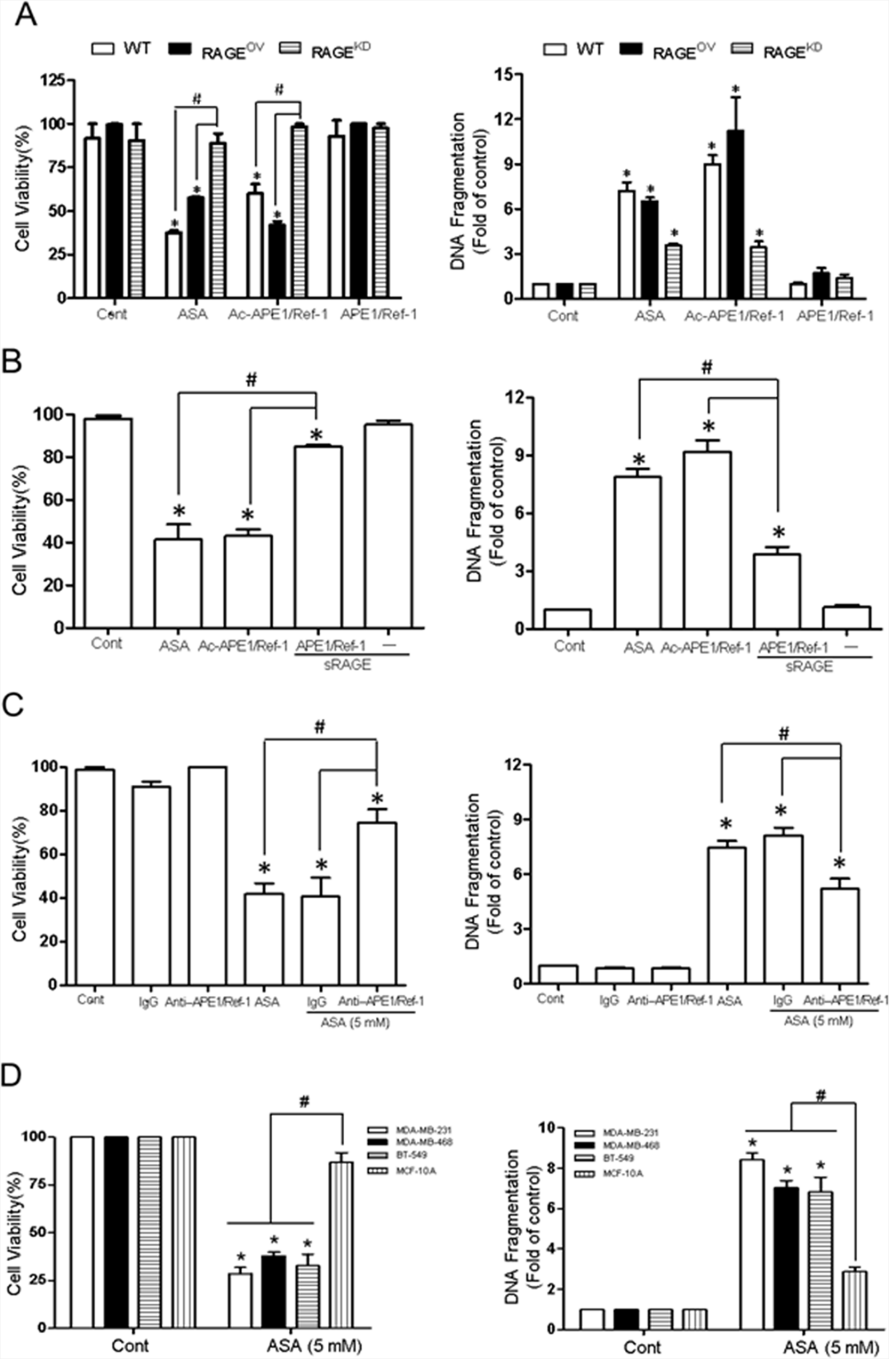
Interaction between RAGE and Ac-APE1/Ref-1 induced apoptotic cell death in TNBCs, leading to a decrease in cell viability **A.** Wild- type, RAGE^OV^, or RAGE^KD^ MDA-MB-231 cells were treated with SA (control), ASA, rh Ac-APE1/Ref-1, or rh APE1/Ref-1. *Columns*, mean (*n* = 3); *bars*, SE. *, ^#^, *P* < 0.05, significantly different compared with corresponding control or within groups by one-way ANOVA followed by Bonferroni's multiple comparison test. **B.** Blocking effects of soluble RAGE or **C.** the anti-APE1/Ref-1antibody on the binding interaction between RAGE and Ac-APE1/Ref-1. Control MDA-MB-231 cells were treated with 5 mM ASA for 6 h to upregulate RAGE. The medium was then replaced with fresh medium. Cells were further incubated with rh Ac-APE1/Ref-1 for 36 h following pretreatment with soluble RAGE (1 μg), rabbit-IgG (2 μg), or anti-APE1/Ref-1 (2 μg) for 2 h. In B and C, *columns*, mean (*n* = 3); *bars*, SE. *, ^#^, *P* < 0.05, significantly different compared with SA-treated control or between groups by one-way ANOVA followed by Bonferroni's multiple comparison test. **D.** MDA-MB-486, BT-549, and normal mammary epithelial MCF-10A cells were treated with 5 mM ASA in the presence of TSA. Cell viability and cytoplasmic histone-associated DNA fragmentation were determined. In A to D, *columns*, mean (*n* = 3); *bars*, SE. *, ^#^, *P* < 0.05, significantly different compared with corresponding SA-treated control cells by paired *t-*test or within-group by one-way ANOVA followed by Bonferroni's multiple comparison test.

Treatment of RAGE^OV^ cells with ASA or rh Ac-APE1/Ref-1 markedly decreased viability (Fig. 7A). In contrast, this treatment did not cause significant death in RAGE^KD^ cells. Consistent with the cell viability results, the cytoplasmic histone-associated DNA fragmentation induced by exposure to ASA or rh Ac-APE1/Ref-1 was significantly increased in RAGE^OV^ cells. The slightly greater magnitude of effects on cell viability and DNA fragmentation in WT cells than RAGE^OV^ cells suggests that treatment with ASA may induce functional alterations of cell-death associated proteins by hyperacetylation [23]. However, exposure to rh APE1/Ref-1 did not affect cell viability or apoptotic DNA fragmentation in RAGE^OV^ cells (Fig. 7A). In RAGE^KD^ cells, cell death was not observed, even though ASA treatment is known to induce apoptosis in cancer cells. These results provided additional evidence for the involvement of Ac-APE1/Ref-1 in the initiation of apoptotic signaling through RAGE.

Because treatment of MDA-MB-231 cells with ASA or rh Ac-APE1/Ref-1 caused DNA fragmentation, a key feature of apoptosis, we proceeded to confirm the role of RAGE in cell death by applying soluble RAGE. The ability of ASA or rh Ac-APE1/Ref-1 to decrease cell viability and induce DNA fragmentation in MDA-MB-231 cells was completely blocked by pre-incubation with soluble RAGE (Fig. 7B). Cytoplasmic histone-associated DNA fragmentation was partially, but significantly, attenuated by blocking RAGE-mediated signaling. The soluble form of RAGE did not have any appreciable effect on cell viability or DNA fragmentation in response to treatment with Ac-APE1/Ref-1.

The ASA-mediated decrease in cell viability was recovered to 80% by removal of Ac-APE1/Ref-1 using an anti-APE1/Ref-1 antibody. In addition, the level of cytoplasmic histone-associated DNA fragmentation was significantly decreased to 33% of the control level (Fig. 7C). These results clearly demonstrate the importance of the binding interaction between RAGE and Ac-APE1/Ref-1 during hyperacetylation-induced apoptosis in MDA-MB-231 cells. Similar to the findings in MDA-MB-231 cells, other TNBC cells (MDA-MB-468 and BT-549) showed decreased viability and increased apoptosis compared with MCF-10A cells in response to hyperacetylation (Fig. 7D).

## DISCUSSION

In the present study, we observed that Ac-APE1/Ref-1 was secreted and acted as a signaling molecule for apoptosis by binding to RAGE in ASA-treated TNBC cells. This conclusion was supported by the following findings: 1) hyperacetylation by co-treatment with ASA and TSA induced different types of cell death in two human breast cancer cell lines (MCF-7 and MDA-MB-231); 2) hyperacetylated MDA-MB-231 cells secreted Ac-APE1/Ref-1 through the formation of vesicles; 3) hyperacetylation upregulated RAGE in MDA-MB-231 cells, which was accompanied by direct binding to the secreted Ac-APE/Ref-1; and 4) RAGE transduced intracellular apoptotic signaling in hyperacetylated TNBC, but not normal breast epithelial MCF-10A cells.

Nuclear PTMs cause dynamic changes that govern transcription-associated genome instability. The association of PTMs with biological processes, especially some PTMs on the histone tail, has been reported in various disease states. PTMs of intracellular proteins can affect enzyme activity and localization, in addition to protein-protein interactions. These effects can modulate various signaling cascades, eventually influencing the extracellular environment. Elucidation of the role of PTMs in cellular events may enable PTMs to be used as tools to sensitize the chemotherapeutic response in cancerous cells, and not just as endogenous regulators of gene expression. The acetylation-dependent regulation of cellular responses may be used to enhance therapeutic responses in the treatment of cancer. For example, acetoxycoumarin is selectively toxic to human lung cancer and glioma cells [43, 44]. In addition, the calreticulin-mediated acetylation system, which uses polyphenolic acetates and acetyl CoA as acetyl group donating molecules, is utilized for the treatment of breast cancer [45]. These studies strongly support the potential therapeutic application of acetylating agents for treatment of cancer.

The present study revealed that Ac-APE1/Ref-1 signaled through autocrine or paracrine pathways to induce apoptotic death of TNBC cells. Under hyperacetylated conditions (mediated by ASA treatment), the nuclear protein APE1/Ref-1 was acetylated and then secreted. The acetylated form of APE1/Ref-1 could be sustained without loss of the acetyl group. Ac-APE1/Ref-1 may thus function similarly to cytokines. For example, HMGB1, which is released in response to treatment with alkylating agents, activates multiple cell surface receptors including TLR2, TLR4, and RAGE, thus functioning as a major *in vivo* sensor of tissue damage by eliciting inflammatory reactions [26]. It is possible that Ac-APE1/Ref-1 initiates an intracellular signaling cascade by binding with modified moieties, i.e., acetyl groups, on RAGE. We also found that the binding of Ac-APE1/Ref-1 to RAGE triggered apoptotic signaling through activation of p38 MAPK, eventually leading to downregulation of Bcl-2 protein, activation of caspases, and PARP-1 cleavage. Previous studies have shown activation of p38 MAPK and ERK after RAGE activation in neuronal diseases [33, 42]. However, further studies are required to determine what signaling molecules are involved in the RAGE-mediated apoptotic signaling pathway. Although RAGE has a short cytoplasmic tail, which is essential for RAGE-mediated cell signaling, this signaling is insufficient to transmit messages directly from an upstream regulator to downstream targets. Thus, signals may be amplified by the involvement of other intracellular molecules.

An alternative activation pathway of RAGE involves NF-κB which controls the transcription of DNA and regulates cellular responses to various stimuli. Inappropriate NF-κB activity has been associated with inflammatory and autoimmune diseases, improper immune development, and cancer. Therefore, in cancer therapy using nonsteroidal anti-inflammatory drugs (NSAIDs) including ASA that have pro-apoptotic activity, effects on the regulation of NF-κB are very important, although still controversial. Some studies have suggested that NSAID treatment has significant anti-tumor effects in gastric, colon, and breast cancers [36, 37, 46]. These reports suggest that ASA-induced apoptosis could be mediated through inhibition of NF-κB. Although NF-κB signaling is a major pathway that activates signaling downstream of RAGE, binding of RAGE to Ac-APE1/Ref-1 did not appear to involve NF-κB activation in the ASA-treated MDA-MB-231 cells (data not shown). Our findings that RAGE was upregulated and that soluble RAGE significantly inhibited apoptosis of TNBC cells by blocking the interaction between acetylated APE1/Ref-1 and RAGE, supports a key role for RAGE in apoptosis that is distinct from the role of NF-κB. Collectively, our experimental data support the premise that the Ac-APE1/Ref-1-RAGE axis is an important target for therapeutic intervention in TNBCs.

Hyperacetylation induced apoptotic cell death was also observed in MDA-MB-468 and BT-549 cells, two other TNBC cell lines. Cell death in these ASA-treated cells was also accompanied by upregulation of RAGE and differed from ASA-treated MCF-7 cells that underwent primarily necrotic death, in which the levels of RAGE were not significantly changed (data not shown). We conclude that the different modes of death between TNBC cells and ER/PR-positive breast cancer cells cannot simply be explained by their differential hyperacetylation-mediated responses. Rather, it is possible that the difference results from the existence of different cell death mechanisms that may involve the secretion of signaling molecules, continuous stimulation of the signaling through autocrine or paracrine pathways, and upregulation of the respective receptors.

In conclusion, the results of the present study indicate that extracellular Ac-APE1/Ref-1 is a potent signaling molecule in hyperacetylated TNBC cells and promotes apoptosis. Additionally, the upregulation of RAGE in response to hyperacetylation mediates apoptosis by direct binding with Ac-APE1/Ref-1. Furthermore, a new understanding of acetylating agents for chemotherapy sensitization to treat drug resistant cancer cells such as TNBCs can be proved preclinically *in vivo*.

## MATERIALS AND METHODS

### Cell culture and acetylation

Human breast adenocarcinoma cell lines (MDA-MB-231, MDA-MB-468, BT-549, and MCF-7) and an immortalized and non-tumorigenic normal human mammary epithelial cell line (MCF-10A) originated from the American Type Culture Collection. Also see Supplementary Materials and Methods. For hyperacetylation of intracellular proteins, cells were treated with 0.1 μM TSA for 1 h followed by different concentrations of ASA for various times. Salicylic acid (SA) was used as a control.

### Preparation of acetylated rh APE1/Ref-1

Purified rh APE1/Ref-1 protein was prepared as described previously [47]. This protein (100 μg) was modified by adding 10 μl of acetic anhydride solution every 1 h (final concentration 17.5 mM), and then incubating for 4 h at pH 7.5. Zeba desalting spin columns (Thermo Fisher Scientific, Waltham, WA, USA) were used to remove unreacted acetic anhydride. The acetylation of APE1/Ref-1 was analyzed by SDS-PAGE and immunoblotting.

### Stable RAGE-over-expressing and -knockdown cell lines

MDA-MB-231 cells were transfected with pcDNA4/TO/myc-his-RAGE (ABGENT, San Diego, CA, USA) or RAGE short hairpin RNA (shRNA) constructs (Santa Cruz Biotechnology, Santa Cruz, CA, USA) to create tet-inducible RAGE overexpressing (RAGE^OV^) and knockdown (RAGE^KD^) lines, respectively. Cell selection was achieved using zeocin or puromycin (Invitrogen Life Technologies, Carlsbad, CA, USA) for overexpression or knockdown cells, respectively. After the selection period, stability of the cell lines was confirmed by immunoblot analyses. The expression of RAGE was maintained constitutively in the presence of doxycycline.

### Determination of cell viability and apoptosis

The effect of hyperacetylation on the viability of MDA-MB-231, MDA-MB-468, BT-549, MCF-7, and MCF-10A cells was determined using propidium iodide (PI) staining and an automatic cell counting machine (ADAM) [27]. Hyperacetylation-mediated apoptosis was quantitatively measured based on DNA fragmentation using a sandwich-type Cell Death Detection ELISA kit (Roche Applied Science, IN, USA) as described previously [48, 49]. In addition, the amount of cells in the sub- G_0_/G_1_ fraction and the fraction of cells stained positively for Annexin V/7-amino-actinomycin (7-ADD, BD Biosciences, USA) in control and hyperacetylated cells were analyzed by flow cytometry as described previously [49]. See also Supplementary Materials and Methods.

### Immunoblotting

Whole cell lysates were prepared and subjected to SDS-PAGE and immunoblotting [27] as described in the Supplementary Materials and Methods.

### Immunoprecipitation

Ac-APE1/Ref-1 in the culture supernatant or whole cell lysates was immunoprecipitated using anti-APE1/Ref-1 antibody, followed by immunoblotting using anti-acetyl-lysine antibody (Cell Signaling Technology, Beverly, MA, USA). For rapid and specific pull-down of acetylated proteins, anti-acetyl lysine agarose beads (ImmuneChem Pharmaceuticals, Canada) and a monoclonal antibody for specific detection of APE1/Ref-1 (N-terminal 80–100 aa, Novus Biologicals, Littleton, CO, USA) were used in some experiments. For details of immunoprecipitation, see Supplementary Materials and Methods.

### Preparation of secretory vesicles

Conditioned medium that was used to maintain MDA-MB-231 cells in the presence of ASA, was collected and centrifuged for 10 minutes at 800 *g* to remove cell debris. Vesicles were enriched by sequential centrifugation [50]. See also Supplementary Materials and Methods.

### Fractionation to obtain plasma membranes

See Supplementary Materials and Methods.

### Binding of Ac-APE1/Ref-1 to RAGE

The binding interaction between RAGE and Ac-APE1/Ref-1 was assessed by co-immunoprecipitation of secreted Ac-APE1/Ref-1 from conditioned medium, the membrane fraction expressing RAGE, and rh proteins Ac-APE1/Ref-1 or RAGE-Fc. See also Supplementary Materials and Methods.

### Proximity ligation assay (PLA)

The binding between RAGE and Ac-APE1/Ref-1 was visualized using a Duolink II fluorescence kit (Sigma-Aldrich) according to the manufacturer's protocol as described in Supplementary Materials and Methods.

### Immunocytochemistry

MDA-MB-231 cells were cultured on coverslips and treated with SA or ASA for 6 h in the presence of TSA. For plasma membrane staining, permeabilization was omitted. Immunofluorescence staining was performed as described previously [49]. Also see Supplementary Materials and Methods.

### Transmission electron microscopy (TEM)

To visualize secretory vesicles containing Ac-APE1/Ref-1, TEM was performed as described previously, with some modifications [48]. See Supplementary Materials and Methods.

### Statistical analyses

All statistical tests were performed using GraphPad Prism v.5.01 (La Jolla, CA, USA) software. Statistical significance of differences in measured variables between control and treated groups was determined by paired *t*-test or one-way ANOVA followed by Dunnett's or Bonferroni's multiple comparison tests. Differences were considered significant at *P* < 0.05.

## SUPPLEMENTARY MATERIALS AND METHODS


