# Supplementary material for: Extracellularly secreted APE1/Ref-1 triggers apoptosis in triple-negative breast cancer cells via RAGE binding, which is mediated through acetylation
Source: Oncotarget. 2015 Jun 23;6(27):23383–98. doi: 10.18632/oncotarget.4345 (PMC4695125; doi:10.18632/oncotarget.4345)
Supplement: Supplementary file 1 [file oncotarget-06-23383-s001.pdf]

SUPPLEMENTARY MATERIALS AND METHODS

REAGENTS

Acetylsalicylic acid (ASA), salicylic acid (SA), TSA, sodium butyrate, nicotinamide, and the Duolink PLA assay kit were purchased from Sigma-Aldrich (St. Louis, MO, USA). Dulbecco's modified Eagle's medium, RPMI-1640, fetal bovine serum, antibiotics mixture, phosphate buffered saline (PBS), trypsin, and CellMask deep red plasma membrane stain were purchased from Invitrogen Life Technologies (Carlsbad, CA, USA). Recombinant human RAGE-Fc and soluble RAGE were from R&D system (Minneapolis, MN, USA) and ProSpec (Rehovot, Israel), respectively. Antibodies against RAGE, CD-9, GAPDH, and Bax were from Santa Cruz Biotechnology (Santa Cruz, CA, USA). β-actin and P-cadherin antibodies were from Sigma-Aldrich and Abcam (Cambridge, MA, USA), respectively. Antibodies against phospho-p38 MAPK, phospho-Erk, phospho-JNK, caspase-3, and acetyl-lysine were from Cell Signaling (Danvers, MA, USA). Antibodies against poly (ADP-ribose) polymerase-1 (PARP-1) and Bcl-2 were from BD Biosciences (San Jose, CA, USA). An agarose conjugated antibody specific for acetyl-lysine was from ImmuneChem Pharmaceuticals (Burnaby, BC, Canada). Two different APE1/Ref-1 antibodies were used. A monoclonal antibody for detection of APE1/Ref-1 (N-terminal 80–100 aa) was obtained from Novus (Littleton, CO, USA). A polyclonal APE1/Ref-1 antibody was generated by immunizing rabbits with recombinant human APE1/Ref-1 (rh APE1/Ref-1) and purified by affinity chromatography. The RAGE overexpression construct, and RAGE short hairpin RNA (shRNA) plasmid were purchased from ABGENT (San Diego, CA, USA) and Santa Cruz Biotechnology, respectively.

CELL CULTURE

Human breast adenocarcinoma cell lines (MDA-MB-231, MDA-MB-468, BT-549, and MCF-7) and an immortalized and non-tumorigenic normal human mammary epithelial cell line (MCF-10A) were originated from the American Type Culture Collection. MCF-7 cells and BT-549 cells were maintained in Dulbecco's modified eagle's medium (DMEM) (Gibco, Grand Island, NY, USA) supplemented with 10% fetal bovine serum (FBS), 0.01 mg/ml human recombinant insulin and 1% penicillin/streptomycin. MDA-MB-468 cells were maintained in Leibovitz's L-15 medium (Gibco, Life Technologies) supplemented with 10% FBS, MDA-MB-231 cells were cultured in RPMI 1640 (Gibco) supplemented with 10% FBS and 50 μg/ml gentamycin (Gibco). MCF-10A cells were cultured in Mammary Epithelial Cell Growth Medium (MEGM™ Bullet Kit, Lonza, Inc., Basel, Switzerland) supplemented with 10% FBS.

DETERMINATION OF APOPTOTIC CELL DEATH

Quantification of DNA fragmentation was done using a sandwich-type ELISA, which determines cytoplasmic histone-associated DNA fragments by the activation of endonucleases. The effect of hyperacetylation on DNA fragmentation was determined using a kit according to the manufacturer's recommendations. Briefly, cells were plated, preincubated with TSA, and exposed to SA or ASA for the indicated times and concentrations. After acetylation, floating and attached cells were separately collected and processed for analysis of necrosis or apoptotic DNA fragmentation using an anti-histone antibody in 96-well plates. The histone–DNA complexes were recognized by anti-DNA-peroxidase antibodies and the level of binding was quantified by measuring the colorimetric reaction at 405 nm. The analysis of necrotic or apoptotic cell death induced by treatment with ASA was also performed by flow cytometry. The acetylated cells were collected at different times and analyzed for apoptosis following double staining with Annexin V-PE and 7-AAD (7-amino-actinomycin D) as indicated by the manufacturer (BD Biosciences). Analysis was performed on FACScan cytometer (BD Biosciences) using CellQuest software.

IMMUNOBLOTTING

Proteins were separated by sodium dodecyl sulphate-polyacrylamide gel electrophoresis (SDS-PAGE), transferred to polyvinylidene difluoride membrane After transfer, the membrane was incubated with appropriate primary and secondary antibodies and the immunoreactive bands were detected using the enhanced chemiluminescence method [27]. In some experiments, each membrane was stained with Ponceau S to confirm the same amount of protein was present before incubation with the primary antibody. Stripping and re-probing was performed for some proteins to normalize for differences in transfer and protein loading. Antibodies against RAGE, CD-9, GAPDH, and Bax were from Santa Cruz Biotechnology. β-actin and P-cadherin antibodies were from Sigma-Aldrich (St. Louis, MO, USA) and Abcam (Cambridge, UK) respectively. Antibodies against phospho-p38 MAPK, phospho-Erk, phospho-JNK, caspase-3, and acetyl-lysine were from Cell Signaling (Danvers, MA, USA). Antibodies against poly (ADP-ribose) polymerase-1 (PARP-1) and Bcl-2 were from BD Biosciences.

IMMUNOPRECIPITATION

Ac-APE1/Ref-1 in the culture supernatant or whole cell lysates was immunoprecipitated using anti-APE1/Ref-1 antibody. One microgram of anti-APE1/Ref-1 was added to the culture supernatant and incubated for 2 h at 4°C. Protein A/G agarose beads were then added to each sample and the incubation was continued for 16 h. The immunoprecipitated complexes were washed twice with buffer containing 50 mM Tris-HCl (pH 7.5), 1% Triton X-100, 5% glycerol, protease and phosphatase inhibitor cocktails, 10 mM nicotinamide, 10 mM sodium butyrate, and 5 µM TSA. The immune complexes were mixed with sample buffer and subjected to SDS-PAGE followed by immunoblotting using anti-acetyl-lysine antibodies. Anti-acetyl-lysine antibody is specific for acetylated proteins.

PREPARATION OF SECRETORY VESICLES

Conditioned medium, that was used to maintain MDA-MB-231 cells in the presence or absence of ASA, was collected and centrifuged for 10 minutes at 800 *g* to remove cell debris. Vesicles were enriched by sequential centrifugation steps [50]. After passing through a 0.2-µm pore size filter, secretory vesicles in the medium were obtained by centrifugation at 100, 000 *g* for 210 min. The vesicles were washed, re-centrifuged, suspended in 100 µl PBS, and analyzed for APE1/Ref-1 content followed by SDS-PAGE and immunoblotting.

FRACTIONATION TO OBTAIN PLASMA MEMBRANES

Cells were suspended in buffer A (250 mM sucrose, 1 mM EGTA, 10 mM HEPES, protease inhibitor, pH 7.5) and homogenized for 10 seconds. After centrifugation for 15 min at 500 *g*, buffer B (0.1 M Na_2CO_3) was added to the supernatant and incubated with shaking for 45 min at 4°C. The sample was centrifuged for 15 min at 100,000 *g*, the pellet suspended in buffer C (250 mM sucrose, 1 mM $MgCl_2$, 10 mM HEPES), and analyzed for RAGE by SDS-PAGE and immunoblotting.

BINDING OF AC-APE1/REF-1 TO RAGE

The binding interaction between RAGE and Ac-APE1/Ref-1 was assessed by co-immunoprecipitation of secreted Ac-APE1/Ref-1 from conditioned medium, the membrane fraction expressing RAGE, and the rh proteins Ac-APE1/Ref-1 or RAGE-Fc (R&D system, Minneapolis, MN, USA). RAGE-Fc protein (1 µg), immunoprecipitated by protein A/G agarose beads, was incubated with Ac-APE1/Ref-1 in binding buffer (20 mM Tris-HCl, pH 7.4, 0.1% Triton X-100, 5% glycerol, 10 mM butyrate, 10 mM nicotinamide, 5 µM TSA) and stirred at 4°C overnight. The RAGE-Fc protein was reacted with conditioned medium and collected at the indicated times. Alternatively, APE1/Ref-1 immunoprecipitated from conditioned medium was incubated with RAGE protein. The immunoprecipitated APE1/Ref-1 was also incubated with the membrane fraction from ASA-treated cells. The protein immune complexes were washed four times with binding buffer. The bound proteins were eluted with 2 × reducing SDS loading buffer, resolved by SDS-PAGE, and immunoblotted.

PROXIMITY LIGATION ASSAY (PLA)

The binding between RAGE and Ac-APE1/Ref-1 was visualized using a Duolink II fluorescence kit (Sigma-Aldrich). MDA-MB-231 cells grown on glass coverslips were treated with SA or ASA for 12 h. After removal of medium, the cells were reacted for 3 h with rh Ac-APE1/Ref-1 or culture supernatant containing secreted Ac-APE1/Ref-1. In some experiments, $RAGE^{OV}$ MDA-MB-231 cells were incubated with rh Ac-APE1/Ref-1. Cells were fixed with PBS containing 4% paraformaldehyde for 15 min. For staining of RAGE in the plasma membrane, permeabilization was not performed. To reduce non-specific signals, cells were incubated with a blocking solution for 60 min, and then with a mixture of mouse anti-RAGE monoclonal antibody (1:100, Santa Cruz) and anti-APE1/Ref-1 rabbit polyclonal antibody (1:100) overnight at 4°C. To determine *in situ* whether the proteins were within 40 nm proximity, specific conjugated oligonucleotides (PLA probe anti-mouse minus and anti-rabbit plus) were added and incubated for 1 h at 37°C. The negative control was performed by adding only one primary antibody (RAGE mouse monoclonal) to the cells. Fluorescent microscopic images of the cells were acquired using ex/em of 590/670 nm for the PLA signal and 410/470 nm for DAPI (Karl Zeiss, Oberkochen, Germany). For all multicolor imaging, signals were acquired sequentially.

IMMUNOCYTOCHEMISTRY

MDA-MB-231 cells were cultured on coverslips and treated with SA or ASA for 6 h in the presence of TSA. The cells were then washed with PBS, fixed with 4% paraformaldehyde, and permeabilized with 0.1% Triton X-100. For plasma membrane staining, permeabilization was omitted. Immunofluorescence staining was performed as described previously [49]. Cells were incubated with primary antibodies (APE1/Ref-1, 1:200; RAGE, 1:100) for 1 h at room temperature. After immunofluorescence staining, cells were counterstained

with DAPI (10 ng/mL) for 10 min. The cells were visualized under a confocal microscope.

TRANSMISSION ELECTRON MICROSCOPY (TEM)

To visualize secretory vesicles containing Ac-APE1/Ref-1, TEM was performed as described previously, with some modifications [48]. Briefly, MDA-MB-231 cells (2×10^5/well) were treated with either SA or 5 mM ASA for 6 h at 37°C. The cells were harvested and fixed in ice-cold electron microscopy grade 2.5% glutaraldehyde. The specimens were rinsed, postfixed, dehydrated in a graded ethanol series, and embedded in epoxy resin. Ultrathin sections (78 nm) were cut, attached onto copper grids, and stained with a rabbit anti-APE1/Ref-1antibody followed by an anti-rabbit IgG antibody conjugated to 15-nm gold particles (Cytodiagnostics, Ontario, Canada). The grids were post-stained with droplets of uranyl acetate and lead citrate. To assess the specificity of the anti-APE1/Ref-1antibody, additional spirochete grids were processed as described above without the primary antibody. The sections were imaged using TEM (Hitachi, Tokyo, Japan) at 7, 000× and 30, 000× magnifications.
